# Exploring *RNF213* in Ischemic Stroke and Moyamoya Disease: From Cellular Models to Clinical Insights

**DOI:** 10.3390/biomedicines13010017

**Published:** 2024-12-26

**Authors:** Benjamin Y. Q. Tan, Charlene H. P. Kok, Megan B. J. Ng, Shaun Loong, Eric Jou, Leonard L. L. Yeo, Weiping Han, Christopher D. Anderson, Chiea Chuen Khor, Poh San Lai

**Affiliations:** 1 Division of Neurology, Department of Medicine, National University Hospital, Singapore 119074, Singapore; meganng@nuhs.edu.sg (M.B.J.N.); leonard_ll_yeo@nuhs.edu.sg (L.L.L.Y.); 2 Department of Medicine, Yong Loo Lin School of Medicine, Singapore 117597, Singapore; e0500915@u.nus.edu; 3 Institute of Molecular and Cell Biology (IMCB), Agency for Science, Technology and Research (A*STAR), Singapore 138632, Singapore; weiping_han@imcb.a-star.edu.sg; 4 Faculty of Medicine, Imperial College London, London SW7 2AZ, UK; charlene.kok22@imperial.ac.uk; 5 Department of Oncology, University of Oxford, Oxford OX3 7DQ, UK; eric.jou@oncology.ox.ac.uk; 6 Program in Medical and Population Genetics, Broad Institute of MIT and Harvard, Cambridge, MA 02142, USA; cdanderson@mgb.org; 7 Department of Neurology, Brigham and Women’s Hospital, Boston, MA 02115, USA; 8 Genome Institute of Singapore (GIS), Agency for Science, Technology and Research (A*STAR), Singapore 138632, Singapore; khorcc@gis.a-star.edu.sg; 9 Department of Paediatrics, Yong Loo Lin School of Medicine, National University of Singapore, Singapore 119228, Singapore; paelaips@nus.edu.sg

**Keywords:** stroke, moyamoya, *RNF213*, genetics, intracranial stenosis, intracranial atherosclerotic disease

## Abstract

Advances in stroke genetics have highlighted the critical role of rare genetic variants in cerebrovascular diseases, with *RNF213* emerging as a key player in ischemic stroke and Moyamoya disease (MMD). Initially identified as the primary susceptibility gene for MMD, *RNF213*—notably the p.R4810K variant—has been strongly linked to intracranial artery stenosis (ICAS) and various ischemic stroke subtypes, particularly in East Asian populations. This gene encodes an E3 ubiquitin ligase with diverse roles in angiogenesis, vascular remodeling, lipid metabolism, and cerebral blood flow regulation, yet its exact mechanisms in cerebrovascular pathology remain incompletely understood. This review synthesizes findings from genetic studies, as well as cellular and animal models, to provide a holistic understanding of *RNF213*’s involvement in cerebrovascular diseases. Key mechanisms by which *RNF213* variants contribute to disease pathogenesis are explored, alongside discussions on their clinical utility as biomarkers and therapeutic targets. Additionally, we address the gene’s implications for disease prediction, risk assessment, and cascade screening. By integrating evidence across disciplines, this review identifies critical knowledge gaps, including the biological pathways underlying *RNF213*’s pathogenicity. These insights lay the groundwork for future research and underscore the potential of *RNF213* in driving personalized approaches to cerebrovascular disease management.

## 1. Introduction

Ischemic stroke, affecting over 7 million people annually, remains a leading cause of death and long-term disability [1]. Although there have been significant advances in identifying cardiometabolic risk factors such as hypertension, diabetes and atrial fibrillation, the underpinning genetic factors for ischemic stroke have not been well established, despite a high degree of heritability of up to 37.9% [2]. Although genome-wide association studies (GWASs) have identified several common variants associated with stroke risk, recent studies have identified pathogenic low-frequency genetic variants, which may have larger effect sizes and provide crucial insights into stroke pathophysiology [3,4].

Among these rare variants, those in the *RNF213* gene have emerged as particularly intriguing. Initially identified in 2011 as the major susceptibility gene for Moyamoya disease (MMD), a rare cerebrovascular disorder characterized by progressive stenosis of intracranial arteries, *RNF213* has since been implicated in various subtypes of ischemic stroke [5,6]. This gene encodes an E3 ubiquitin ligase with critical roles in angiogenesis [7], vascular remodeling [8], lipid metabolism [9] and cerebral blood flow regulation [10]. However, its precise function in stroke pathogenesis remains elusive [11].

Emerging evidence suggests that *RNF213* variants may influence stroke risk by precipitating a broader spectrum of vascular conditions, including intracranial artery stenosis (ICAS) [12]. The p.R4810K (p.Arg4810Lys, c.14429G>A) variant, in particular, has been associated with an increased risk of ICAS in East Asian populations [12,13]. These findings highlight the potential of *RNF213* as both a biomarker and therapeutic target for intracranial artery stenosis and stroke, underscoring the need for more in-depth and comprehensive understanding of its role in cerebrovascular pathology.

This narrative review explores the current understandings of *RNF213*’s multifaceted role in ischemic stroke and MMD. Evidence encompassing a broad range of sources, including cellular and animal models and clinical observations across diverse populations, is interrogated in detail, and the potential mechanisms by which *RNF213* variants contribute to disease are discussed. In particular, the clinical implications of these findings for risk assessment, prevention, and treatment strategies are highlighted. By bridging human population genetic studies with basic science research, this review provides a comprehensive perspective on *RNF213*’s significance in ischemic stroke and MMD and outlines critical areas for future research.

## 2. Clinical Insights of *RNF213*

### 2.1. RNF213: The Main Susceptibility Gene for Moyamoya Disease

*RNF213* (Ring Finger Protein 213; Mysterin) is the main susceptibility gene for MMD, a rare, progressive ischemic cerebrovascular disorder [14]. MMD is characterized by progressive, non-atherosclerotic stenosis and occlusion of the carotid arteries, particularly affecting the terminal portion of the intracranial internal carotid artery (ICA), proximal portions of the middle cerebral artery (MCA), and anterior cerebral artery (ACA) [14]. As a compensatory mechanism to reduced blood flow, a fragile vascular network of vessels, termed moyamoya vessels, forms near the stenotic or occlusive arterial lesions [15]. These vessels have a smoke-like appearance on angiographs, therefore being called moyamoya, which is a Japanese word that is an onomatopoeia that translates to “puff of smoke”. The stenosis and occlusion of intracranial vessels predispose those affected by MMD to ischemic stroke, while the fragile moyamoya vessels are prone to rupture, resulting in further risk of hemorrhagic stroke.

Although the exact cause of MMD is unknown, MMD is much more common in those with East Asian ancestry (especially Japan, Korea, and China) [16], and a positive family history is observed in 9–15% of patients [15,17]. Furthermore, MMD has been associated with many genetically transmitted disorders such as Trisomy 21 and neurofibromatosis type 1, altogether indicating strong genetic underpinnings. This led to an unbiased genome-wide linkage analysis performed in 2008 by Mineharu et al. on 15 extended Japanese MMD families, which mapped the disease to a region on the long arm of chromosome 17 (17q25.3) [18]. Subsequently, in 2011, two independent Japanese studies identified *RNF213* as the culprit gene for MMD, which exhibits an autosomal dominant inheritance pattern with incomplete penetrance [5,19].

### 2.2. RNF213 *p.R4810K*, the Founder Variant in Moyamoya Disease

The *RNF213* gene is large (591 kD, 67 coding exons, and 5207 amino acids) [20] and harbors multiple pathogenic missense variants that have been implicated in MMD. *RNF213* p.R4810K (c.14576G>A) is the MMD founder variant with allele frequencies of up to 0.283% among East Asian populations [6]. It is found in around 95% of patients with familial MMD [19] and 70–90% of sporadic Japanese and Korean MMD patients [21]. A meta-analysis of 2353 MMD cases and 5488 controls demonstrated a strong association between *RNF213* p.R4810K and MMD in Japanese, Korean, and Chinese populations, with odds ratios (ORs) of 184.04, 109.77, and 31.53, respectively [22]. In a meta-analysis involving three Japanese population studies with 46,958 East Asian individuals (17,752 cases and 29,206 controls), *RNF213* p.R4810K was associated with increased stroke risk (OR 1.91, 95% CI 1.55–2.36). This association was more pronounced in women, and the mean age of stroke onset was 4.1 years earlier in carriers than in non-carriers [23].

Recent studies suggest that *RNF213* p.R4810K may be a prognostic marker for surgical outcomes in MMD patients [24]. Those without this variant tend to have a lower incidence of collateral vessel development from extracranial arteries [25], a reduced likelihood of favorable outcomes following indirect revascularization [24], and a decreased risk of prolonged hyperperfusion after direct revascularization surgery [26]. These findings highlight the potential impact of *RNF213* p.R4810K on vascular adaptation and clinical prognosis in MMD.

The zygosity of the *RNF213* p.R4810K variant also has direct implications on clinical phenotypes. Homozygous carriers for the p.R4810K variant have a higher risk of developing early-onset MMD, with median ages of onset at 3 years for homozygous and 7 years for heterozygous carriers [27]. These individuals also present with more severe symptoms at diagnosis, including cerebral infarction, a more extensive distribution of stenosis and occlusion (involving posterior cerebral arteries and both hemispheres), and poorer long-term prognosis, such as cognitive impairment [28]. This suggests that the zygosity of the p.R4810K genotype could serve as a valuable biomarker for identifying patients at risk of early-onset or more severe MMD, potentially guiding decisions for earlier interventions such as bypass surgery. Furthermore, incorporating this genetic information into cascade screening and monitoring strategies could enhance early detection and improve disease management in affected individuals. A study employing systematic molecular karyotyping, exome sequencing, and automated structural assessment of missense variants in a cohort of 88 MMD patients identified a strong association between *RNF213* pathogenic variants and distinct phenotypic differences. Based on these findings, the authors recommend MRI-MRA screening for asymptomatic siblings carrying the pathogenic variant and highlight the crucial role of genetic testing, including cascade screening, in facilitating early detection and proactive management strategies [29].

The incomplete penetrance of MMD seen in carriers of the *RNF213* p.R4810K variant raises the possibility of an environmental trigger. Studies suggest that microbial infections could serve as such a trigger in individuals with this *RNF213* polymorphism, given the gene’s crucial role in antimicrobial defense, which may be aberrantly activated during infections and inflammation [30]. Furthermore, epidemiological studies suggest that MMD occurs in clustered regions, with leptospirosis accounting for specific clusters [31]. This supports the idea that bacterial infections at various stages of life could initiate vascular remodeling, helping to explain the variable clinical presentation of MMD. On the other hand, the potential role of viral infections in the pathogenesis of MMD remains controversial. A cross-sectional study including 111 MMD patients suggested that vasculopathy-related viruses, including varicella-zoster, measles, rubella, mumps, Epstein-Barr, herpes simplex virus, cytomegalovirus, human parvovirus B19, human herpesvirus 6, human herpes virus 8, and John Cunningham virus, are unlikely to have an impact on MMD development [32]. However, this contradicts a study that suggests that Epstein-Barr viruses might be involved in the pathogenesis of MMD [33]. The lack of studies exploring this association raises the possibility that the role of viruses or environmental triggers could be underexplored rather than absent. Additionally, the correlations could reflect dysfunctional metabolic responses instead, especially with studies suggesting that *RNF213* expression is upregulated by inflammatory cytokines such as interferon-β and interferon-γ [34], both of which are induced in viral infections. Future studies that employ larger cohorts are needed to clarify this relationship.

Caution should also be exercised when considering *RNF213* p.R4810K as a screening or diagnostic tool in clinical practice. While this variant is associated with a significantly increased risk and occurs in around 1–2% of the East Asian population [12], only 1/150–1/300 of heterozygous carriers actually develop MMD [28]. In contrast, homozygous carriers of the variant exhibit a much higher incidence rate, exceeding 78% [27].

### 2.3. Other RNF213 Variants in Moyamoya Disease

According to gnomAD v4.1.0 data, approximately 17,700 variants have been identified among 1,500,009 alleles in the *RNF213* gene. Of these, there are 4 alleles identified as pathogenic, namely p.D4013N, p.S4118N, p.S4118P, and p.R4131C, and 12 likely-pathogenic alleles (p.N38K, p.L4010V, p.H4014N, p.C4017Y, p.C4017S, p.C4020F, p.K4115del, p.P4119S, p.F4120L, p.D4122A, p.K4185E, p.V4941E) [35]. Most of these variants are also classified as pathogenic or likely pathogenic in ClinVar [36], a public database of variants with interpretation of disease significance and supporting evidence. A meta-analysis revealed that two rare variants, p.E4950D and p.A5021V, increased the risk of MMD in the Chinese population significantly, with an odds ratio of 9.1 and 5.0, respectively [22]. Furthermore, sequencing of *RNF213* has uncovered additional novel variants in both East Asian and Caucasian cases, though the mechanistic role of these variants in MMD remains unclear [22,28].

**Figure 1:**
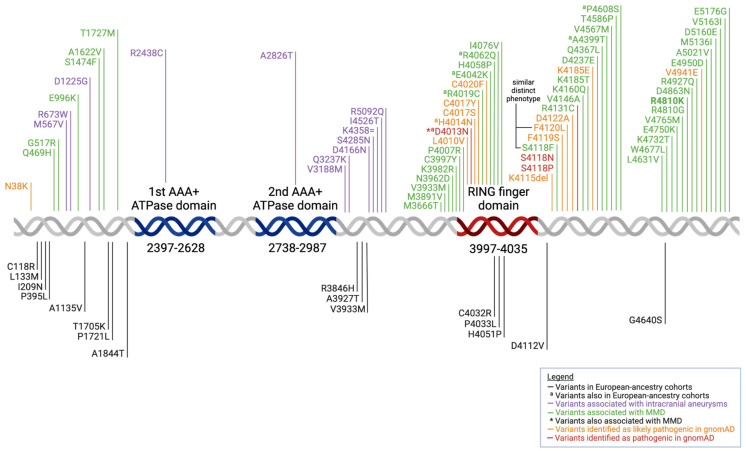
Schematic representation of the *RNF213* protein and its variants identified. The domain structures based on Morito et al. [39]. Variants are organized as likely pathogenic (in orange) or pathogenic (in red) in gnomAD [35], variants found in European-ancestry patients (in black or marked ^a^) [40], variants associated with MMD (in green) or intracranial aneurysms (in purple) [38].

Certain *RNF213* variants have been associated with distinct phenotypes. For instance, patients with the p.F4120L substitution have displayed a severe syndromic form of MMD similar to that of patients carrying the p.S4118F substitution [37]. Both of these amino acid substitutions occur in a highly conserved region near the RING domain of the *RNF213* protein. These patients present with a triad of severe, very early-onset MMD; occlusion of other arteries, including the abdominal aorta; and liver susceptibility with recurrent elevated aminotransferases [37]. In general, mutations within the RING finger domain are related to MMD, while mutation sites in the AAA+ (ATPases Associated with diverse cellular Activities) ATPase domain are related to IA [38] (details shown in Figure 1). Not only that of point mutations, frameshift mutations in *RNF213* are also associated with aneurysms (c.1214_1216delGAG and c.11415delC), MMD (c.1587_1589delCGC and c.12343_12345delAAA), and cerebrovascular disease (c.1214_1216delGAG and c.11415delC).

It has also been shown that an *RNF213* variant with a higher functional effect on the gene could lead to a more severe form of MMD. Patients with a compound heterozygous variant (*RNF213* p.R4810K variant together with other rare variants) exhibited a more severe form of the disease characterized by younger onset, posterior cerebral artery involvement, and a higher percentage of presentation with infarction, as compared to patients with the heterozygous *RNF213* p.R4810K variant alone [27].

Two GWAS studies of MMD have further identified several common *RNF213* variants associated with the risk of disease [41,42]. Of note, the *RNF213* c.97+1955T>C variant was associated with early-onset (*p* = 4.57 × 10^−54^; OR = 1.96) as opposed to late-onset MMD (*p* = 0.003) [42]. This implicates common *RNF213* variants in the pathophysiology of MMD and raises the possibility of building polygenic scores to delineate the genetic risk of MMD.

### 2.4. RNF213 in Europeans

Although the incidence of MMD is much higher in East Asia as compared to that in Europe [28], *RNF213* variants have also been identified in European MMD patients and are a recognized susceptibility factor for various cardiovascular conditions [43], such as hypertension and extracerebral artery stenosis.

Clinical phenotypes are seemingly distinct between East Asian and European MMD patients, with the latter typically displaying a less severe form of MMD. European-ancestry MMD patients have a lower rate of hemorrhage, with 8.5% presenting with intracranial hemorrhage [28] compared to 25.3% in Asia [44], and a greater proportion harbor unilateral disease compared to Asian patients (17% in Europe [45] as compared to 10.6% of MMD patients in Japan [46]).

In European-ancestry MMD patients, the missense variants are preferentially clustered in the C-terminal region, which is either within or close to the region encoding the RING finger domain [40]. However, no major founder variant is identified. This is contrasted to the East Asian population, where *RNF213* p.R4810K is the founder variant, but this variant has never been detected in European-ancestry patients [40]. This is relevant because *RNF213* variant diversity has been shown to be associated with different phenotypes, and the difference in *RNF213* mutations between ethnicities could explain the different severity of MMD observed. The *RNF213* p.R4810K mutation has been shown to be the main cause of ICAS and the MMD ischemic phenotype, while the *RNF213* p.A4399T mutation is related to the MMD bleeding phenotype [38]. Furthermore, mutations in the RING finger domain have been linked to MMD, while mutations in the AAA+ ATPase domain have been linked to intracranial aneurysms with an increase in ATPase activity [47]. This leads to speculations that the mutations cause an increase in ATPase activity to promote angiogenesis, contributing to the formation of intracranial aneurysms [38]. Hence, this suggests that different mutation sites may be involved in different mechanisms. However, comprehensive studies mapping the specific regions of the *RNF213* gene to its specific molecular function have yet to be performed.

### 2.5. RNF213 Involvement in the Different Phenotypes of MMD

**Figure 2:**
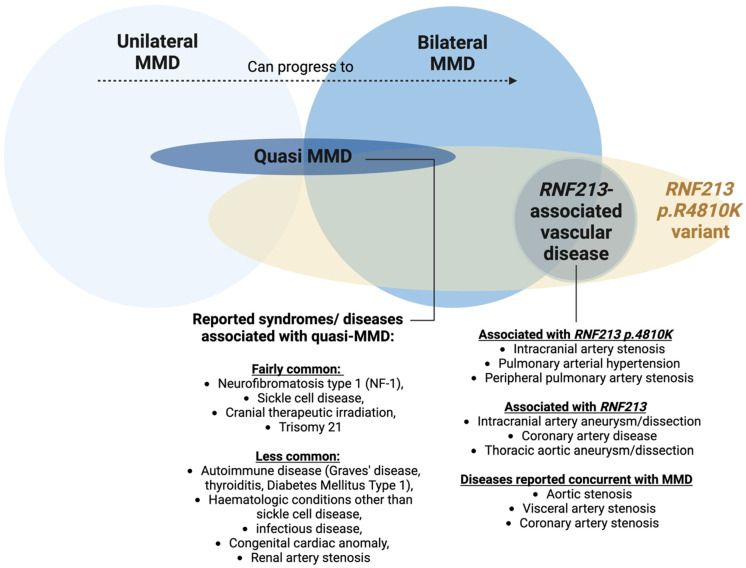
Schematic illustrating the relationship between unilateral MMD, bilateral MMD, quasi-MMD (Moyamoya syndrome), *RNF213*-associated vascular disease, and the p.R4810K variant. The figure also includes a list of syndromes and diseases associated with quasi-MMD and *RNF213*-associated vascular disease. Image created using BioRender.

The diagnosis of MMD hinges on angiographic findings, where stenosis or occlusion is observed in the arteries at the terminal portion of the intracranial internal carotid artery, accompanied by the presence of Moyamoya vessels—abnormal vascular networks near the occlusive or stenotic lesions [48]. MMD can present as either unilateral or bilateral, with the progression from unilateral to bilateral involvement being highly common in young patients [49]. It is essential to exclude other secondary conditions to make a diagnosis of MMD [50]. These conditions include autoimmune diseases such as systemic lupus erythematosus, antiphospholipid antibody syndrome, polyarteritis nodosa, and Sjögren syndrome. In addition, meningitis, Trisomy 21, neurofibromatosis type 1, sickle cell disease, and cerebrovascular lesions resulting from head irradiation must also be considered [51,52]. Should any of these associated conditions be present, the diagnosis is classified as quasi-MMD (or Moyamoya syndrome) (Figure 2). Quasi-MMD differs from MMD in that it occurs in the context of other conditions and may also involve the posterior circulation, which is not typically seen in MMD [49].

The *RNF213* p.R4810K variant is strongly associated with both unilateral and bilateral MMD, with an odds ratio of 54.0 for unilateral MMD and 144.0 for bilateral MMD, respectively [53]. The identification of this variant as a common risk factor for both imaging phenotypes has led the Research Committee on Moyamoya Disease (Spontaneous Occlusion of the Circle of Willis) in Japan to officially recognize both unilateral and bilateral MMD as part of the same disease entity [46].

A prospective study on 18 quasi-MMD and 91 controls in Japan by Morimoto et al. suggested that the frequency of p.R4810K carriers was significantly higher in quasi-MMD disease cases than in controls (OR = 89.0) [54]. However, the association between the variant and quasi-MMD varies depending on the underlying associated disease. The strongest association was observed between the p.R4810K variant and autoimmune-related quasi-MMD, as highlighted by a study in a Chinese cohort of 42 patients and 161 controls, which found a significant association of the variant with both autoimmune and atherosclerotic quasi-MMD [55]. Both atherosclerosis and autoimmune diseases are driven by inflammatory processes, and research has shown that proinflammatory cytokines such as tumor necrosis factor-alpha (TNFα) and interferon-gamma (IFNγ) can synergistically induce *RNF213* expression, leading to angiogenesis in vitro and in vivo [56]. Several other studies have also reported an association between autoimmune hyperthyroidism (such as Graves’ disease) and the p.R4810K variant, with elevated antithyroid antibodies linked to MMD [57,58,59]. Although the exact mechanism is unknown, it is hypothesized that high levels of thyroid antibodies may damage vascular walls by modulating their sensitivity to the sympathetic nervous system [58].

An association between the p.R4810K variant and quasi-MMD, associated with disorders that have mutations in the Ras signaling pathway, such as neurofibromatosis type 1 (NF1) patients and Noonan syndrome, has also been found, although to a lesser degree than that of the autoimmune associations [54]. In the case of NF1 quasi-MMD, Phi et al. observed that 18.7% of patients with NF1 carried the p.R4810K variant, and that harboring the p.R4810K variant is particularly associated with bilateral disease [60]. Mechanistically, the Ras signaling pathway is known to play critical roles in stimulating the inflammatory response [61], which may explain the similar association observed to that of p.R4810K and atherosclerotic and autoimmune quasi-MMD. Accordingly, others have found that NF1 patients have increased levels of pro-inflammatory cytokines IL-6 and IL-1β and a number of inflammatory monocytes [62,63]. Alternatively, the downstream proliferative effect of the Ras signaling pathway may also explain the association between NF1 and quasi-MMD [54,64]. NF1 is a condition characterized by the loss of neurofibromin, which is the product of the causative gene for NF1 [65]. Deficiency in neurofibromin triggers abnormal proliferation of vascular endothelial cells and intimal hyperplasia of arteries after vascular injury, as shown in in vitro knockdown studies and in vivo NF-1 knockdown mouse models [66]. This leads to aberrant vascular morphogenesis, with a vasculopathy compatible with the definition of MMD [67], and studies have also indicated that the p.R4810K variant is associated with bilateral involvement in NF1 quasi-MMD [60].

Overall, the p.R4810K variant is suggested to confer susceptibility to quasi-MMD, especially that of atherosclerosis and autoimmune-related quasi-MMD etiologies, and hence could have the potential to be used to identify at-risk individuals. However, larger studies are needed to confirm these findings.

### 2.6. RNF213-Associated Vascular Disease

Although *RNF213* p.R4810K is most commonly associated with MMD, this variant has also been linked to other vascular diseases, such as ICAS [22,53], peripheral pulmonary artery stenosis, and pulmonary arterial hypertension [68]. Other heterozygous *RNF213* mutations have also been observed in some vascular diseases, such as intracranial artery aneurysm [47] and dissection [69], thoracic aortic aneurysm and dissection [70], and coronary artery disease [71]. These vascular diseases have similar pathogenesis, where they all involve progressive stenosis and subsequent occlusion, and can occur concurrently in patients with *RNF213* mutations, termed *RNF213*-associated vascular disease [72].

Although related vasculopathies are not well characterized in MMD patients, several findings point towards *RNF213* having a greater involvement in vasculopathy beyond that of MMD. Firstly, a gene-dosage effect has been described for MMD and systemic vascular diseases [73]. Those heterozygous for the *RNF213* p.R4810K variant are more likely to be associated with classical isolated MMD or being an asymptomatic carrier. On the contrary, being homozygous for the variant is associated with both MMD and systemic vascular diseases [74], with a unique pattern of diffuse narrowing across the entire aorta together with coronary, renal, iliofemoral, splanchnic, and peripheral pulmonary arterial stenosis [75]. Secondly, histopathologic studies revealed similar characteristics between the various associated vasculopathies—the proliferation of the smooth muscle cells is the cause of the arterial occlusion in MMD and is also part of the mechanism of atherosclerosis in coronary heart disease [76,77], for which *RNF213* p.R4810K is also an identified risk locus [43]. This points towards *RNF213* playing a role in the progressive narrowing and occlusion of vessels beyond that of MMD. Elucidating the mechanism that causes vasculopathy could help design more rational therapeutics. Furthermore, coronary heart disease (CHD) tends to have significant mortality and morbidity [78]; hence, understanding the association is important to elucidate if screening tests should be performed to reduce these risks.

The main vessels affected concurrently with MMD in homozygous *RNF213* p.R4810K patients are the aorta, visceral, and coronary arteries [74]. In these patients, the aorta shows distinct pathology characterized by diffuse widespread narrowing throughout its length with no signs of vascular inflammation, with the most significant constriction occurring in the infra-renal abdominal segment [74]. This is again gene-dose dependent, with the aorta in heterozygous patients only being affected to a mild degree, and the infrarenal abdominal aorta is generally unaffected [75]. It is also distinct from other diseases with aortic involvement, such as Williams syndrome and other forms of aortitis, where the stenosis occurs in different parts of the aorta. This distinct pattern is also observed in visceral arteries, where the affected vessels are those of the superior mesenteric, celiac, and renal arteries, with diffuse ostial stenosis that is also gene-dose dependent (heterozygous patients’ visceral arteries are rarely affected) [75]. Homozygous patients generally do not develop mesenteric or abdominal ischemia as these patients typically have patent inferior mesenteric arteries that provide collateral circulation [74]. Accordingly, MMD has also been shown to be associated with coronary artery disease, and MMD patients may have an increased risk of myocardial infarction after coronary artery bypass grafting [79].

Similar to MMD, mutations in *RNF213* show incomplete penetrance of developing *RNF213*-associated vascular disease [75], and furthermore the underlying factors that determine which organs or vessels are affected remain unclear. Currently, it is thought that environmental factors, epigenetics, or additional disease-modifying genetic loci may be involved in determining the vascular phenotypes [80,81,82]. Accordingly, vascular endothelial cell-specific expression of mutant *RNF213* in transgenic mice aggravated the development of pulmonary hypertension after exposure to hypoxia [80], suggesting that *RNF213* variants may interact with environmental triggers such as hypoxia and trigger the onset of specific vascular phenomena. Hypoxia and other environmental factors, such as infectious triggers, are known to induce epigenetic changes [81,82], and whether these affect variant *RNF213* expression and the nature of the underlying interactions remains to be elucidated.

### 2.7. RNF213: A Tool to Guide the Misclassification of MMD as ICAS?

The three main diagnostic criteria for MMD on magnetic resonance angiogram (MRA) are (1) stenosis/occlusion of the terminal intracranial ICA, (2) bilateral distribution of the first finding, and (3) abnormal vascular network in the basal ganglia signifying the presence of ‘moyamoya’ vessels [48]. Although ICAS is characterized by atherosclerotic narrowing of the intracranial arteries, whereas MMD differs in that it is a non-atherosclerotic process, both diseases can present similarly on MRA or computed tomography angiogram, which are the main imaging modalities used currently [83]. This may potentially lead to misclassification of MMD as ICAS or vice versa. This is especially so when there are no objective criteria for abnormal basal collateral vessels, the extent of it being less prominent in adult-onset MMD, and that it might not be observed in the early stages of MMD [84]. Furthermore, characteristic MMD findings are not consistently observed at all stages of progression of MMD or between patients [14,84,85], and patients may have stenotic lesions in the MCA or ACA with a relatively non-occluded distal ICA in early stages of MMD [84] or a purely unilateral disease.

In a two-center-based case-control study in Japan with 323 patients, *RNF213* p.R4810K was significantly associated with non-MMD ICAS with an odds ratio of 16.8 [86]. A different study of 352 patients showed that the variant was observed in half of ICAS patients and further demonstrated that the frequency of *RNF213* protein-altering variants was higher with an increasing number of angiographic criteria observed—out of all the patients that met all three of the MMD diagnostic criteria, 75.6% had the variant, 57.7% of patients met two criteria, and 28.6% of patients met one criterion [84]. This, coupled with the absence of quantifiable criteria for angiogram-based MMD diagnosis, suggests that some MMD patients may have been misclassified as having intracranial atherosclerosis or ICAS [84]. High-resolution vessel wall MRI might help differentiate between the characteristic vessel wall shrinkage seen in MMD and the atherosclerotic plaques typically observed in ICAS [87]. Distinguishing between MMD and ICAS is critical, as their management strategies differ significantly. While symptomatic MMD may be managed with surgical revascularization, ICAS treatment mainly involves medical therapy with antithrombotics, statins, and intracranial stenting in refractory cases [88]. Furthermore, whilst MMD can lead to both ischemic and hemorrhagic strokes, ICAS typically causes ischemic stroke as a result of thrombosis, hypoperfusion, or perforator disease in the absence of neovascularization [89]. An ongoing clinical trial is investigating whether there is a proportion of ICAS-diagnosed patients who have MMD instead through follow-up angiography and systematic testing for genetic biomarkers, elucidating whether *RNF213* variants may have a future role in distinguishing between ICAS and MMD [90].

*RNF213* has also been reported to be a susceptibility gene for non-cardioembolic stroke and ischemic stroke attributable to large artery atherosclerosis [23]. In the meta-analysis that included 46,958 individuals of East Asian ancestry, 5.2% of patients with non-cardioembolic stroke had the *RNF213* p.R4810K variant, with an odds ratio of 2.60 [23]. When analyzed for the ischemic stroke subtype, large artery atherosclerosis was found to be significantly associated with the variant [23].

The identification of candidate genetic variants associated with specific stroke subtypes, such as MMD or ICAS, could pave the way for a novel genetic diagnostic framework. Previously, some of these conditions were thought to be primarily driven by acquired risk factors [91]. This genetic insight could lead to earlier detection and preventative care for stroke patients who carry the relevant alleles. Potential strategies might include regular neurovascular imaging for surveillance, as well as targeted interventions like intensive blood pressure and cholesterol management, which could help mitigate the risk of stroke occurrence in at-risk individuals.

## 3. Role of *RNF213* in Cellular Pathways: Functional Studies

**Figure 3:**
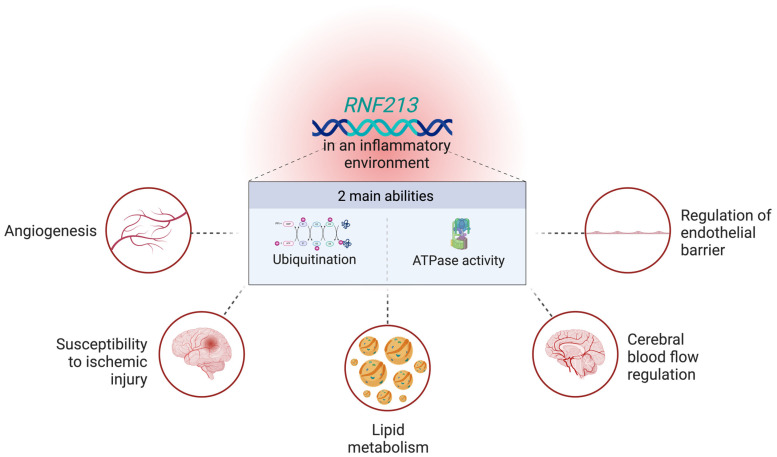
Roles of *RNF213* in cellular pathways in an inflammatory context.

Although *RNF213* has been identified as the major susceptibility gene for MMD in Asian populations [5], the mechanisms in which *RNF213* mutations affect the pathophysiology of MMD have yet to be elucidated. This complicates efforts to pinpoint potential therapeutic targets for MMD treatment. At present, *RNF213* mutations have been implicated in a broad range of biological pathways such as lipid metabolism [9], angiogenesis [7], regulation of the endothelial barrier [8], susceptibility to ischemic injury [10], as well as cerebral blood flow regulation [10] (Figure 3). Indeed, many of these pathways have been shown to be associated with ischemic stroke [3].

### 3.1. Molecular Structure of RNF213 and Its ATPase and Ubiquitinase Activity

The *RNF213* gene is ubiquitously expressed throughout the body in humans and mice [92] and is found in tissues throughout the body [93]. Its function and role in cellular pathways are mainly due to its ubiquitin ligase and ATPase activities, and it is unique in being the only known protein that exerts both activities [9]. Although it has a large molecular size (591 kD), only a few domain structures on the *RNF213* gene have been determined, namely the AAA+ ATPase module and a single RING finger ubiquitin ligase domain, and its functions are linked to these domains [39]. AAA+ proteins exert their activity by coupling chemical energy derived from ATP hydrolysis to translocate or remodel macromolecules in an energy-dependent process [94]. Functions of known AAA+ proteins are diverse and include protein degradation, DNA recombination, replication and repair, as well as bacteriochlorophyll biosynthesis [95]. Ubiquitin ligases are proteins that recruit a ubiquitin carrier, recognize a protein substrate, and assist or directly catalyze the transfer of the ubiquitin from the carrier to the substrate [96]. This results in the proteolysis or functional regulation of the substrate [96]. *RNF213* has been shown to exert a ubiquitylation activity both towards a variety of substrate proteins, as well as itself, in a process known as autoubiquitylation [9].

### 3.2. Role of RNF213 in Lipid Metabolism and Mediating Lipotoxicity

*RNF213* is a positive regulator of cellular fat storage and primarily increases lipid droplets (LDs) through modulating the lipolytic process and eliminating adipose triglyceride lipase, the rate-limiting lipase from LDs [9]. This results in lipid droplet stabilization to allow storage of neutral lipids, thereby preventing lipid-induced cellular stress while maintaining energy reserves and lipid homeostasis [9]. Hence, in MMD-associated mutations with a loss-of-function mutation in the RING finger ubiquitin ligase domain of *RNF213,* there is significant impairment of this fat-stabilizing activity and detachment from LDs, as well as the formation of an amorphous aggregate-like pattern [9]. Similarly, knockdown of endogenous *RNF213* also shows significant fat loss in cells [9]. This is consistent with clinical findings where atherosclerotic changes are not typically found in MMD [15]. Multiple MMD mutations in the RING finger domain of *RNF213* suggest a potential link between the pathogenesis of MMD and dysfunction of LDs, and hence could be a particularly important perspective for future research.

Furthermore, *RNF213* knockdown has a role in protecting against lipotoxicity induced by palmitate, a saturated fatty acid, and palmitate-induced cell death [97]. *RNF213* knockdown also normalized downstream effects of lipotoxicity by regulating palmitate-induced changes in gene expression (blocking the induction of endoplasmic reticulum (ER) stress genes) and reducing the splicing of *XBP1* mRNA during palmitate exposure (which is a messenger RNA that is triggered by the accumulation of unfolded proteins in the ER) [97].

Although the most direct effect of targeting lipid droplets appears to be that of metabolic regulation, recent findings suggest that this could also play a role in cell-autonomous immunity [30]. Lipid droplets have been identified as a key component of the innate immune response, accumulating and assembling multiple host defense proteins with antimicrobial properties on their surface when triggered by bacterial infection or lipopolysaccharide (LPS) stimulation [30]. Although no direct link to antimicrobial function has been established, *RNF213* is one of the proteins that accumulate on the surface in response to both interferon and LPS stimulation [30].

### 3.3. Role of RNF213 in Angiogenesis and Vascular Remodeling

*RNF213*-deficient brain endothelial cells generated using CRISPR-Cas9 exhibited angiogenic features similar to MMD, such as enhanced and disordered angiogenesis [98]. Similar knockdown studies performed in human umbilical vein endothelial cells suggested that the disrupted angiogenesis was due to the downregulation of DNA replication and proliferation pathways and the sensitization of endothelial cells to LPS-induced inflammation. This leads to slowed cell migration and increased macrophage transmigration [7]. Interestingly, *RNF213* knockdown in the absence of an inflammatory environment did not influence endothelial migration but rather enhanced the retardation of LPS-induced inflammatory migration [99]. This suggests that the disrupted angiogenesis pathology seen in the MMD context is associated with inflammation [99].

Furthermore, *RNF213* influences endothelial cell-to-cell communication as well as changes at a transcriptional level and alternative splicing level with vascular smooth muscle cells [99]. This could explain the MMD angiographic phenotype of the thinning of the tunica media [100]. The transcriptomic changes observed are similar to datasets from proteome analysis of exosomes in MMD patients [101], suggesting that the observed changes in MMD patients could be of vascular origin. This is corroborated in in vivo *RN213* knockout mice studies, where the morphological examination of cerebral vessels observed irregularities in vessel diameter, enhanced vessel tortuosity, and a decrease in microvessel density [5,102]. This suggests impairment in the vascular network consistent with MMD patients due to abnormal angiogenesis and vascular remodeling processes.

*RNF213* deficiency also induces nuclear factor κB (NF-κB) activation [103], which has a central role in being the signal integrator that controls vascular inflammation. In particular, it does so through monocyte activation via the IL-6 pathway, which has multiple roles in initiating and sustaining vascular inflammation [103]. This corroborates literature that examined plasma samples from 20 MMD patients against 9 controls and revealed an increased concentration in matrix metalloproteinases, monocyte chemoattractant protein-1, pro-inflammatory cytokines (TNF-α, IL-1ß, IL-6), and vascular endothelial growth factor [104]. Regulating the levels of proteins that promote or inhibit angiogenesis ensures balance in pro- or anti-angiogenic signals, maintaining proper angiogenic responses.

*RNF213* knockdown in human brain microvascular endothelial cells also activates the Hippo pathway effectors, Yes-associated protein (YAP) and transcriptional coactivator with PDZ-binding motif (TAZ), resulting in the overexpression of the downstream effector vascular endothelial growth factor receptor 2 (VEGFR2) [100]. VEGFR2 regulates the formation of blood vessels, the proliferation and migration of endothelial cells, and is the main receptor for vascular endothelial growth factors (VEGFs), which are the primary regulators of angiogenesis [105]. Furthermore, inhibition of YAP/TAZ reversed the *RNF213* knockdown-induced angiogenesis [100], suggesting that the discovery of a YAP/TAZ inhibitor might represent a therapeutic target for MMD patients with *RNF213* loss-of-function mutations.

### 3.4. RNF213 and Its Involvement in the Regulation of the Endothelial Barrier Integrity

*RNF213* regulates endothelial barrier integrity through increased cell proliferation and migration, corroborating the angiogenic phenotype seen in MMD patients with fibrocellular concentric hyperplasia of the tunica intima due to proliferation of smooth muscle cells and extracellular matrix within the intima [98]. The increased cell proliferation of endothelial cells occurs through the increased expression of cell cycle-promoting genes in endothelial cells, activation of the PI3K-AKIT pathway (a major driver of protein synthesis and cell growth), and induction of matrix metalloproteinase-1 (MMP1) [7]. Furthermore, ECM receptor-related genes in induced pluripotent stem cells from MMD patients with the p.4810K variant are significantly downregulated, and they produce fewer ECM components compared to healthy controls [34]. These processes occur when *RNF213* is transcriptionally induced by the co-stimulation of pro-inflammatory cytokines, including TNFα [56], implicating that the fibrillogenesis process is linked with an inflammatory context.

*RNF213* also regulates endothelial barrier integrity through increased permeability and downregulation of interendothelial junction proteins [7]. Clear morphological changes and an increased blood-brain barrier permeability were observed in knockdown studies in brain endothelial cells through the downregulation of platelet endothelial cell adhesion molecule-1 (PECAM-1), an essential interendothelial junction protein that has key roles in the blood-brain barrier maintenance [7]. This results in an enhanced ability to transmigrate leukocytes and secrete increased levels of proinflammatory cytokines [106], creating an inflammatory environment to activate angiogenesis. This is corroborated in in vivo studies, where permeability assays in *RNF213*-deficient mice showed higher levels of tracer extravasation into brain tissues [107], emphasizing the dysregulation of the endothelial barrier in the absence of *RNF213*. Chemokines from defective colony-forming endothelial cells could result in pathological recruitment and proliferation of vascular smooth muscle cell progenitors in MMD patients [108].

Taken together, these observations suggest the importance of inflammatory signals as environmental factors and that *RNF213* mutations could be an early pathological mechanism leading to MMD.

### 3.5. RNF213 in Cerebral Blood Flow Regulation

*RNF213* knockout mice exhibit reduced baseline CBF in both cortical and subcortical regions as compared to wild-type mice [10]. In induced ischemia, namely that of bilateral common carotid artery stenosis as seen in MMD patients, the knockout mice showed significantly impaired recovery of cerebral blood flow (CBF) following induced ischemia as compared to wild-type mice [10]. Furthermore, cerebral angiogenesis by hypoxia was suppressed in *RNF213* knockout mice [10], which could contribute to MMD, as the suppression of cerebral angiogenesis would decrease oxygen delivery to the brain and impair stroke recovery [10]. These experiments reveal the crucial role *RNF213* plays in ensuring healthy cerebral vascular function both in the presence and absence of acute ischemic events and the impact of reduced *RNF213* function in impairing the adaptive vascular responses needed to mitigate ischemic damage, leading to a positive feedback loop of greater tissue injury.

## 4. Potential for Translation and Therapeutic Target

The current management for MMD typically involves a combination of medications and bypass surgery [109]. Antiplatelet agents are often prescribed to reduce the risk of ischemic stroke, while bypass surgery may be an option in select cases [109]. However, these treatments do not modify the underlying disease pathogenesis and do not address the root cause of MMD. This highlights the urgent need for disease-modifying therapies.

Understanding the function of *RNF213* and its role in the pathophysiology of MMD could open new therapeutic avenues. Targeting the molecular pathways influenced by *RNF213* may lead to the development of new medications or repurposing existing drugs. For example, YAP/TAX inhibitors could potentially normalize aberrant vascular processes in MMD patients with *RNF213* mutations. Several small molecule inhibitors targeting the YAP/TAZ-TEAD interaction have been developed and are currently being tested in clinical trials for various cancers [110]. Adapting these inhibitors for MMD treatment could provide a novel therapeutic approach. However, it’s important to note that while this strategy may have merit, further research is needed to fully understand the complex interplay between *RNF213* and YAP/TAZ signaling in the context of MMD and to evaluate the efficacy and safety of YAP/TAZ inhibition in this specific patient population.

### Future Directions in Exploring the Biological Role of RNF213

Epidemiological findings strongly suggest a direct involvement of *RNF213* in the onset of MMD [16]. However, the specific pathophysiology and its specific biological role as to how it results in the disease, as well as which regions in this especially large protein elicit its biological functions, are unclear. Given the multi-hit hypothesis discussed above, future research could be directed to identifying and understanding the environmental triggers that seem to correlate with the phenotype in *RNF213* carriers. This could help uncover new opportunities for prevention or early intervention through targeted therapies or lifestyle modifications to mitigate disease risk in vulnerable populations.

The biological functions of *RNF213* shown in the current literature all point towards specific pathological characteristics of MMD—lipid metabolism (could contribute to the stenosis/occlusion of the terminal portion of the carotid arteries), angiogenesis and regulation of the endothelial barrier (formation of the moyamoya vessels), susceptibility to ischemic injury, and cerebral blood flow regulation (impaired recovery post-occlusion of the carotid arteries). From the perspective of MMD research, further exploration of these functions and linking them to specific characteristics of MMD research could help in the understanding of the disease. For example, characterization of the aggregate-like structures observed with the *RNF213* RING finger mutants and exploring their similarities to both cells that are directly affected by MMD mutations and cells that participate in the formation of the stenotic lesion (both of which remain unclear) could help bridge the link between the cellular models and the pathophysiology of MMD. Furthermore, precise dissection of the various regions of the *RNF213* gene, namely the RING finger ubiquitin ligase domain and the AAA+ ATPase module and a single RING finger ubiquitin ligase domain, and evaluating potential regulatory crosstalk of the activity of both regions could help understand if the variety of biological functions is due to direct recognition by the *RNF213* gene with a common downstream molecule or if there are various unique mechanisms that correspond to each target.

## 5. Conclusions

In conclusion, *RNF213* is known to play a role in various intracranial steno-occlusive diseases, including unilateral and bilateral MMD, as well as quasi-MMD. Recent research has also implicated it in the more prevalent intracranial atherosclerotic disease. This gene holds promising clinical applications, such as predicting the onset and severity of disease and aiding in the screening of family members who may benefit from regular neurovascular imaging surveillance. However, several key challenges remain unresolved. Understanding the biological mechanisms underlying the *RNF213* variant’s contribution to disease pathogenesis is essential and could lead to the development of targeted therapies. Additionally, questions remain about whether the p.R4810K variant can serve as a distinguishing marker between ICAS and MMD, potentially guiding screening protocols. Finally, prospective genetic studies on *RNF213* variants are needed to determine its penetrance, expressivity, and variability in clinical outcomes, which will help clarify its role in disease development and patient care.
